# Epileptic seizure classifications using empirical mode decomposition and its derivative

**DOI:** 10.1186/s12938-020-0754-y

**Published:** 2020-02-14

**Authors:** Ozlem Karabiber Cura, Sibel Kocaaslan Atli, Hatice Sabiha Türe, Aydin Akan

**Affiliations:** 1 Department of Biomedical Engineering, Faculty of Engineering and Architecture, Izmir Katip Celebi University, Cigli, Izmir Turkey; 2 Department of Biophysics, Faculty of Medicine, Izmir Katip Celebi University, Cigli, Izmir Turkey; 3 Department of Neurology, Faculty of Medicine, Izmir Katip Celebi University, Cigli, Izmir Turkey; 4 Department of Electrical and Electronics Engineering, Faculty of Engineering, Izmir University of Economics, Balcova, Izmir Turkey

**Keywords:** Electroencephalogram (EEG), Epilepsy, Epileptic seizure classification, Empirical mode decomposition, Ensemble empirical mode decomposition, Intrinsic mode function selection

## Abstract

**Background:**

Epilepsy is one of the most common neurological disorders associated with disruption of brain activity. In the classification and detection of epileptic seizures, electroencephalography (EEG) measurements, which record the electrical activities of the brain, are frequently used. Empirical mode decomposition (EMD) and its derivative, ensemble EMD (EEMD) are recently developed methods used to decompose non-stationary and nonlinear signals such as EEG into a finite number of oscillations called intrinsic mode functions (IMFs). Our main objective in this study is to present a hybrid IMF selection method combining four different approaches (energy, correlation, power spectral distance, and statistical significance measures), and investigate the effect of selected IMFs extracted by EMD and EEMD on the classification. We have applied the proposed IMF selection approach on the classification of EEG signals recorded from epilepsy patients who are under treatment at our collaborator hospital. Multichannel EEG signals collected from epilepsy patients are decomposed into IMFs, and then IMF selection was performed. Finally, time- and spectral-domain, and nonlinear features are extracted and feature sets are created for the classification.

**Results:**

The maximum classification accuracies obtained using various combinations of IMFs were 94.56%, 95.63%, 96.8%, and 96.25% for SVM, KNN, naive Bayes, and logistic regression classifiers, respectively, by using EMD analysis; whereas, the EEMD approach has provided maximum classification accuracies of 96.06%, 97%, 97%, and 96.25% for SVM, KNN, naive Bayes, and logistic regression, respectively. Classification performance with the same features obtained using direct EEG signals instead of the decomposed IMFs was worse than the aforementioned 2 approaches for every combination.

**Conclusion:**

Simulation results demonstrate that the proposed IMF selection approach affects the classification results. Also, EEMD provides a robust method for feature extraction from EEG signals in order to classify pre-seizure and seizure segments.

## Background

Epilepsy is one of the neurological disorders associated with disruption of brain activity that affects approximately 50 million people of the world’s population [1, 2]. Detection of epileptic seizures is performed by neurologists by a visual examination of long-term electroencephalogram (EEG) signals. However, this method is very time-consuming and generally yields incorrect results. On the other hand, epileptic seizures are initiated in different brain lobes of different individuals, so it is not possible to determine a standard focus center for the studies. Therefore long-term EEG recordings are needed to detect epileptic seizures and determine focus center [2–5]. Since visual examination of long-term EEG data makes it difficult to diagnose the disease, automatic seizure detection has become a very popular research area and various signal processing methods have been applied to solve this problem [2, 5, 6].

Many types of seizure detection and classification algorithms have been proposed in the literature [5]. These studies will be briefly discussed in "Related studies" section. In this present study, empirical mode decomposition (EMD) and its derivative, ensemble EMD (EEMD) based classification model for epileptic EEG data is introduced. Our aim is to distinguish pre-seizure and seizure epileptic EEG signals by classifying the features extracted from selected IMFs of EMD, or EEMD. Simulations are performed to evaluate the effectiveness of selecting the IMFs based on some metrics as opposed to using first several IMFs for the classification.

The rest of the paper is organized as follows. The review of some of the previous related work is given in "Related studies" section. Experimental results of the proposed method are shown in "Results" section. Discussion of the results is reported in "Discussion" section. The description of the data set, EMD algorithm, EEMD algorithm and the details of the proposed methodology are discussed in "Materials and methods" section.

### Related studies

Epileptic seizure detection and classification studies have been reported frequently in the literature using various signal processing and classification methods. A variety of features such as temporal, spectral, statistical and nonlinear features are exploited to improve the detection and classification performance.

Several methods have been presented for the detection and classification of seizure and seizure-free EEG segments by using time and frequency domain features such as energy [7], exponential energy [8], matrix determinant [2], spectral power of Hjorth’s mobility components [9], cross-correlation, power spectral density [10], subband spectral powers [11], average value, maximum value, and minimum value [5]. Furthermore, several studies may be found in the literature using the wavelet transform and its derivative approaches [6, 12].

Weighted multiscale Renyi permutation entropy (WMRPE), weighted permutation entropy (WPE), fuzzy entropy (FuzzyEn), a sigmoid entropy, approximate entropy (ApEn) based methods have also been frequently applied to this problem [13–15]. Additionally, nonlinear parameters such as fractal dimension, scaling exponent obtained with detrended fluctuation analysis (DFA), Hurst’s exponent have been utilized in many studies and successful results have been obtained for the detection and classification of seizure and seizure-free epileptic EEG signals [16, 17].

Time–frequency analysis methods such as EMD, EEMD, multivariate empirical mode decomposition (MEMD), complete ensemble empirical mode decomposition (CEEMD) which are developed for the analysis of nonlinear and non-stationary signals, have been successfully applied into detection or classification of seizure and seizure-free epileptic EEG signals in many studies [1, 18–28]. These methods decompose a given signal into a finite number of zero–mean oscillations called intrinsic mode functions (IMFs). One of the major problems while using EMD and other similar decomposition methods is how to choose which IMFs to be used in the classification algorithms. In most studies, the first several IMFs, known to have high-frequency oscillations, are automatically selected for feature extraction [19–22]. It may be discussed that there is a lack of methods in the literature for the selection of best IMFs while using EMD and other similar decompositions.

After a brief investigation, it may be observed that successful classification results have been obtained by using classification algorithms such as support vector machine (SVM) [5, 13–15, 17, 19, 21, 27], Artificial Neural Networks (ANN) [12, 20, 24], K-nearest neighbor (KNN) algorithm [26], Extreme Learning Machine (ELM) [6, 16], Multilayer Perceptron Neural Network (MLPNN) [25], etc.

## Results

EEG signals including pre-seizure and seizure segments obtained from 10-channel EEG recordings of 16 epilepsy patients who are under treatment at Izmir Katip Celebi University School of Medicine, Department of Neurology, were analyzed using EMD, and EEMD approaches and various classifiers. The hybrid IMF selection process including energy, correlation, power spectral distance, and statistical significance measures was carried out for EMD and EEMD approaches in order to identify the IMFs that best represent the original signal as described in "Selection of intrinsic mode functions (IMFs)" section. After the IMF selection process, time-domain (energy, mean value, skewness, and kurtosis) and spectral-domain (total power, spectral entropy, 1st, 2nd, and 3rd moments), and nonlinear (Hurst exponent and Higuchi fractal dimension) feature-sets were created using the selected three IMFs (IMF1, IMF3, IMF2) obtained by EMD, and EEMD approaches, and the EEG signal itself. In addition, we also performed simulations to compare the performance of our proposed approach with that of Discrete Wavelet Transforms (DWT). Since three selected IMFs of EMD and EEMD approaches are used for feature extraction and classification, three-level decomposition is used for DWT utilizing Daubechies4 (db4) mother wavelet function [23]. Finally, SVM, KNN, naive Bayes, and logistic regression classifiers are used for the classification, and the results are evaluated.

Performance evaluation results of our proposed approach are given in Tables 1, 2, 3, 4. In these tables IMF1, IMF2, or IMF3; show that the features for classifications are calculated by using the corresponding IMF; IMF 1–3 denotes that the features are extracted using all three IMFs. On the other hand IMF1– IMF2 shows that the features are extracted from IMF1 and IMF2. Additionally, AC+DC1-3 show that the features are extracted from approximation coefficient (AC) and 3 detail coefficients (DC) of DWT. Furthermore, the italicized numbers in table cells indicate the best performance in accuracy for each approach (Tables 1, 2, 3) and classifier (Table 4).

**Table 1:** Performance results (%) for pre-seizure and seizure EEG signal classification using the time-domain feature-set

Approach	Components	SVM		KNN		Naive Bayes		Logistic regression	
		ACC	*F*-score	ACC	*F*-score	ACC	*F*-score	ACC	*F*-score
EMD	IMF1	55.81	43.64	94.56	94.45	91.56	91.24	94.69	94.94
	IMF2	77.75	79.54	93.50	93.40	91.44	91.06	94.38	94.19
	IMF3	86.19	87.75	93.88	93.75	93.38	93.24	94.69	94.60
	IMF1–IMF2	94.63	94.72	96	95.94	92.25	92.02	93.44	93.42
	IMF1–IMF3	96.12	96.14	95.25	95.19	94.94	94.86	*97.18*	* 97.14*
	IMF2–IMF3	78.63	73.11	94.69	94.60	93	92.84	94.38	94.30
	IMF1–3	74.44	69.07	95.75	95.71	94.19	94.08	96.88	96.88
	IMF1–4	78.88	75.24	95.63	95.54	93.50	93.39	91.56	91.03
EEMD	IMF1	91.38	91.28	95.19	95.20	92.69	92.52	95.31	95.27
	IMF2	62.44	60.80	90.94	90.63	90.63	90.14	92.81	92.60
	IMF3	71.44	69.75	94.44	94.34	93.63	93.55	94.38	94.27
	IMF1–IMF2	96.06	96.04	95.06	95.06	91.75	91.56	95.31	95.30
	IMF1–IMF3	95.50	95.22	96.31	96.28	93.56	93.50	*98.13*	*98.13*
	IMF2–IMF3	92.75	92.33	94.50	94.39	92.38	92.24	94.38	94.30
	IMF1–3	73.44	64.36	96.63	96.61	93.81	93.73	90.94	90.61
	IMF1–4	73.13	68.00	96.50	96.43	92.81	92.71	90.63	90.51
DWT	AC+DC1-3	71.38	60.51	*94.25*	*94.31*	93.50	93.39	92.09	92.06
EEG	All EEG	53.94	45.26	*89.75*	*89.96*	78.94	75.38	87.81	87.21

**Table 2:** Performance results (%) for pre-seizure and seizure EEG signal classification using the spectral feature-set

Approach	Components	SVM		KNN		Naive Bayes		Logistic regression	
		ACC	*F*-score	ACC	*F*-score	ACC	*F*-score	ACC	*F*-score
EMD	IMF1	94.12	94	94.38	94.35	94.56	94.28	85	82.73
	IMF2	94.06	93.81	92.94	92.77	93.75	93.42	94.06	93.97
	IMF3	93.63	93.60	94.75	94.59	95.81	95.66	77.19	80
	IMF1–IMF2	94.69	94.53	93.25	93.15	94.94	94.70	83.13	84.75
	IMF1–IMF3	85.50	86.56	95.44	95.35	*96.88*	*96.77*	94.69	94.50
	IMF2–IMF3	93.34	93.77	94.81	94.66	96.13	95.99	83.44	82.03
	IMF1–3	93	93.31	94.88	94.80	96.19	96.06	82.50	82.93
	IMF1–4	93.81	94.03	94.66	94.59	95.75	95.62	84.38	83.77
EEMD	IMF1	96.06	96.02	95.06	95.05	94.44	94.26	96.25	96.25
	IMF2	92.13	91.90	91.94	91.88	93	92.56	92.50	92.31
	IMF3	94.56	94.48	94.25	94.20	95.56	95.39	96.88	96.86
	IMF1–IMF2	94.38	94.26	94.94	94.88	94.81	94.61	81.88	81.29
	IMF1–IMF3	74.31	73.80	95.31	95.26	96.75	96.6	79.69	78.83
	IMF2–IMF3	94.94	94.83	93.75	93.74	95.69	95.52	*96.88*	*96.89*
	IMF1–3	95.12	94.84	96.69	96.66	96.06	95.93	88.13	88.34
	IMF1–4	91.25	90.64	96.31	96.29	96.81	96.68	90.31	89.42
DWT	AC+DC1-3	81.25	77.56	93.31	93.24	*95*	*94.87*	88.75	88.82
EEG	All EEG	72.06	67.29	*93.31*	*93.37*	77.37	71.59	89.06	88.14

**Table 3:** Performance results (%) for pre-seizure and seizure EEG signal classification using the nonlinear feature-set

Approach	Components	SVM		KNN		Naive Bayes		Logistic regression	
		ACC	*F*-score	ACC	*F*-score	ACC	*F*-score	ACC	*F*-score
EMD	IMF1	80.50	79.17	83.13	82.82	82.69	82.05	83.13	82.80
	IMF2	81.38	80.11	83.31	83.15	86.19	86.79	85.63	85.80
	IMF3	84.75	85.05	81.81	81.52	86.06	86.46	86.25	86.34
	IMF1–IMF2	84.19	83.23	87.31	87.27	88.94	89.18	87.81	87.93
	IMF1–IMF3	88.87	87.85	89.31	89.32	90.75	91.02	89.69	89.72
	IMF2–IMF3	88.19	88.21	86.88	86.93	92	92.32	87.5	87.5
	IMF1–3	90.37	90.15	90.44	90.39	91.81	92.17	90.94	90.97
	IMF1–4	* 95*	*95.01*	93.94	93.86	92.38	92.61	94.38	94.41
EEMD	IMF1	55.88	42.68	59.63	59.05	63.75	56.97	55.63	50
	IMF2	69.73	61.66	79.38	79.47	82.88	84.07	81.25	81.82
	IMF3	70.31	67.75	73.88	73.58	79.88	79.80	77.81	78.15
	IMF1–IMF2	70.06	62.49	77.87	77.63	84.50	84.99	87.50	87.58
	IMF1–IMF3	70.19	66.94	74.19	74.08	80.25	79.65	81.88	81.65
	IMF2–IMF3	77.31	75.39	78.38	78.20	84.50	85.10	83.44	83.28
	IMF1–3	76.69	74.32	78.63	77.69	85.56	85.77	89.06	88.89
	IMF1–4	*92.94*	*92.90*	91.50	91.35	90.69	90.74	91.25	91.19
DWT	AC+DC1-3	64.63	58.12	68.88	67.53	84.50	84.22	* 87.50*	*87.42*
EEG	all EEG	58.19	64.84	67.31	65.83	*69.38*	*68.95*	62.81	65.51

**Table 4:** Performance results (%) for pre-seizure and seizure EEG signal classification using the combined feature-set

Approach	Components	SVM		KNN		Naive Bayes		Logistic regression	
		ACC	*F*-score	ACC	*F*-score	ACC	*F*-score	ACC	*F*-score
EMD	IMF1	94.31	94.16	94.38	94.31	94.31	94.03	86.25	87.06
	IMF2	94.12	93.85	92.62	92.48	93.13	92.79	94.06	94.22
	IMF3	93.38	93.36	94.63	94.45	95.63	95.48	87.50	86.58
	IMF1–IMF2	*94.56*	*94.40*	93.81	93.70	94.56	94.33	92.5	92.31
	IMF1–IMF3	92.06	92.38	*95.63*	*95.53*	*96.88*	*96.77*	*96.25*	*96.23*
	IMF2–IMF3	94.50	94.35	94.81	94.66	95.88	95.74	89.69	89.39
	IMF1–3	90	90.99	94.88	94.81	96.19	96.07	93.75	93.59
	IMF1–4	87.38	85.90	94.63	94.59	96	95.89	92.81	92.55
EEMD	IMF1	*96.06*	*96.04*	94.44	94.43	93.75	93.60	*96.25*	*96.30*
	IMF2	92.44	92.19	91.81	91.69	93.50	93.12	87.19	86.38
	IMF3	94.50	94.42	94.06	94.02	95.44	95.27	92.19	91.80
	IMF1–IMF2	94.94	94.86	94.81	94.76	94.12	93.91	92.50	92.68
	IMF1–IMF3	81.69	80.29	95.94	95.90	* 97*	*96.91*	84.38	84.66
	IMF2–IMF3	94.44	94.32	94.25	94.21	95.38	95.18	91.25	91.36
	IMF1–3	94.19	94.39	*97*	*96.97*	95.75	95.62	90.31	90.22
	IMF1–4	93.56	93.30	96.19	96.17	96.88	96.77	93.13	92.86
DWT	AC+DC1-3	80.81	76.83	93.44	93.38	*94.56*	*94.43*	90.94	90.97
EEG	All EEG	59.75	66.33	*93.25*	*93.35*	78.94	74.41	88.44	87.46

Table 1 summarizes the performance evaluation of time-domain features used for classification. Using the time-domain features calculated from the IMF1–IMF3 (the most favorable two IMFs) of EMD, we obtain 97.18% classification accuracy and 97.14% *F*-score using the logistic regression classifier. While the logistic regression algorithm yields the highest accuracy (98.13%) and *F*-score (98.13%) values by using the time-domain features calculated from IMF1–IMF3 of EEMD, the SVM algorithm performs the worst (ACC: 62.44%, F1-score: 60.80%) for the same features calculated from IMF2. When the same features calculated from the subbands obtained using DWT, we achieved 94.25% accuracy and 94.31% *F*-score for the KNN classifier. To reveal the effect of decomposition, we analyzed the EEG signal itself and repeated the above feature extractions and classification. Using the time-domain features and KNN classifier, we obtain 89.75% accuracy and 89.96% *F*-score, where the SVM performed very poorly (ACC: 53.94% and *F*-score: 45.26%). Results of all classification using time-domain features are provided in Table 1.

We give the performance metrics for spectral features used in classification for different IMF combinations in Table 2. We observe that naive Bayes provides 96.88% accuracy and 96.77% *F*-score using spectral features calculated from IMF1–IMF3 of EMD. However, higher classification performance is obtained by the same features calculated from IMF2–IMF3 of EEMD with logistic regression. While 95% accuracy and 94.87% *F*-score were obtained from the spectral feature of DWT using naive Bayes classifier; 93.31% accuracy and 93.37% *F*-score were achieved using the same feature obtained from EEG signals itself.

Classification results using nonlinear features are given in Table 3. The results suggest that the nonlinear features extracted from IMF1–4 of EMD provided classification performance with 95% accuracy and 95.01% *F*-score using KNN and SVM. However, EEMD approach provided 92.94% accuracy and 92.90% *F*-score using the same features with SVM. Using the features obtained from the EEG signal itself, accuracy and *F*-score are obtained 69.38% and 68.95%, respectively, with KNN. On the other hand, 87.50% accuracy and 87.42% *F*-score were obtained using the nonlinear feature of the DWT approach by the logistic regression classifier.

In order to determine the effect of IMF selection on the classification performance and to compare the approaches, the classification is performed with the combination of time, spectral, and nonlinear features. The classification results are shown in Table 4. In EMD approach, the SVM provided the maximum classification accuracy (94.56%) using combined features of IMF1–IMF2. However, KNN (95.63%), naive Bayes (96.88%), and logistic regression (96.25%) classifiers resulted in the highest accuracies using combined features of IMF1–IMF3.

On the other hand, in the EEMD approach SVM (96.06%) and logistic regression (96.25%) classifiers provided the highest classification accuracy for the combined features of IMF1. While KNN (97.06 %) achieves the best performance using combined features of IMF1–3, naive Bayes (97%) yielded maximum classification accuracy using the combined feature of IMF1–IMF3.

DWT approach provided maximum classification accuracy of 94.56% with naive Bayes classifier for the combined features of subbands. Notice that by using the same features extracted from the EEG signal (the last row), KNN (93.25%) provides the best classification performance. We also observed that the classification performance of the combined feature-set created by using the EEG signal is worse than the EMD and EEMD approaches. Furthermore, the highest classification performance for all classifiers is achieved using features extracted by EEMD approach. Apart from the selected first 3 IMF, the success of the classification was not improved when the features obtained using the 4th IMF were included in the classification process.

**Fig. 1:**
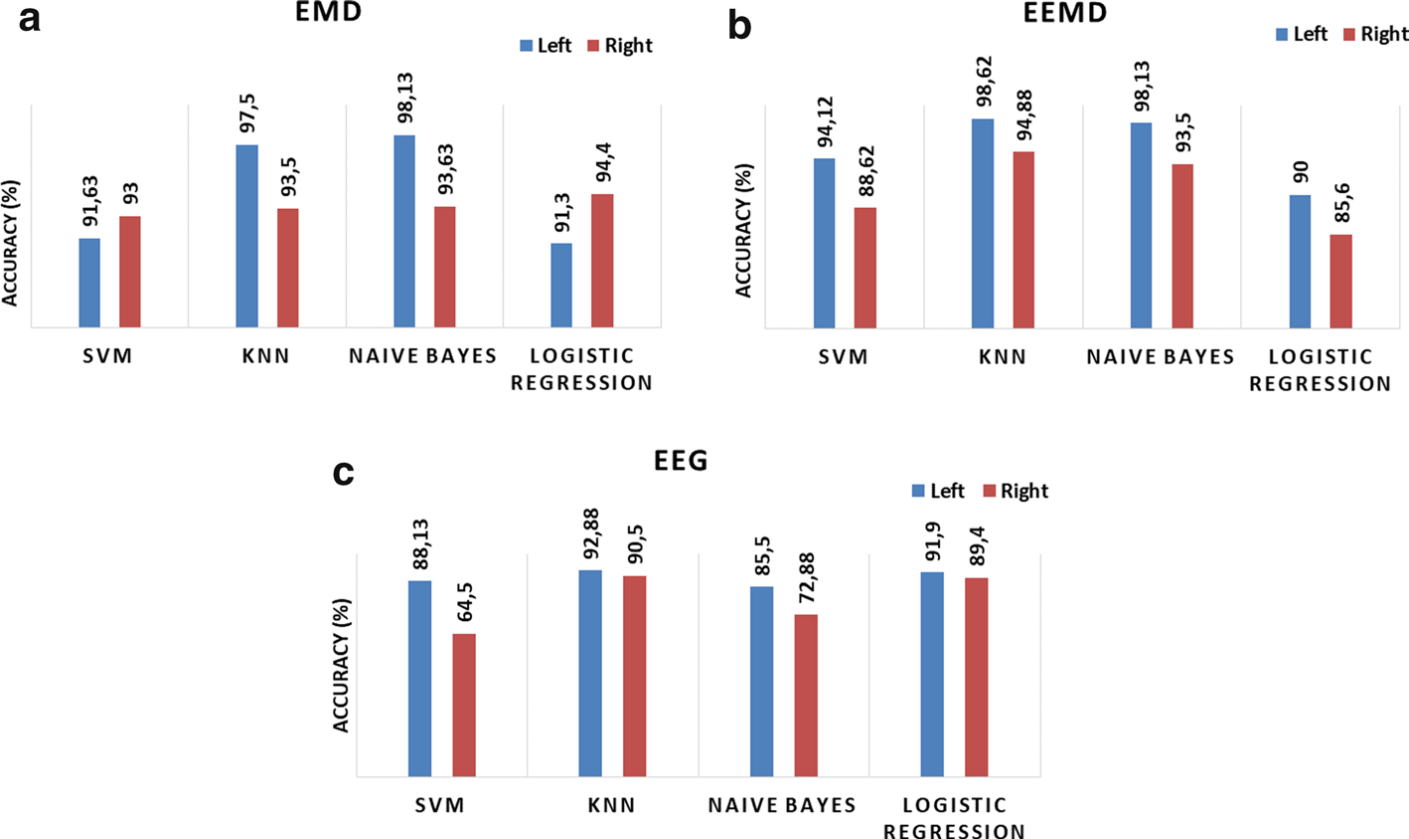
Hemisphere-based mean classification accuracy for **a** EMD approach, **b** EEMD approach, and **c** EEG signals. Here, left and right hemispheres were represented with blue and red, respectively

In order to investigate the channel-based performance of our approaches, the classification is performed for 10 channels separately using total features of IMF1–3. The average mean classification accuracies for the channels in the left (Fp1–F7, F7–T1, T1–T3, T3–T5, Fp1–F3 channels) and right (Fp2–F8, F8–T2, T2–T4, T4–T6, Fp2–F4 channels) hemispheres are calculated. The classification accuracy of EEMD- and EEG-signal based approaches are higher in the left hemisphere for all four classifiers (shown in Fig. 1b, c). These results are supported by the clinical information about epileptic focus areas of patients in our study, shown in Table 5. However, in the EMD-based approach, the classification accuracy is higher for the left hemisphere only for KNN and naive Bayes classifier (shown in Fig. 1a).

**Table 5:** Summary of the EEG data set used in the proposed study

Subjects	Gender	Epileptic focus areas	Age-duration
Patient 1	F	LTemp	
Patient 2	F	LTemp	
Patient 3	F	LTemp	
Patient 4	F	LTemp	
Patient 5	F	LTemp	
Patient 6	M	LTemp	
Patient 7	M	LTemp	
Patient 8	M	RFron.-Temp	Age: 37.3${\mp }$7
Patient 9	M	RFron.-Temp	Duration: 1 min
Patient 10	M	L.Temp	
Patient 11	M	L.Temp	
Patient 12	M	R.Temp	
Patient 13	M	R.Temp	
Patient 14	M	L.Temp	
Patient 15	M	L.Temp.	
Patient 16	M	L.Temp	

*F* female, *M* male, *LTemp* left temporal, *RTemp* right temporal, *RFron.-Temp* right fronto-temporal

## Discussion

In our proposed study, the main objective is to present a hybrid IMF selection method and explore the effect of selected IMFs extracted by EMD and EEMD, on the classification performance. Our approach investigates the advantage of using EEMD, where noise-added versions of the signal are decomposed to eliminate the well-known, mode-mixing problem of EMD. The problem of mode mixing can be described as the occurrence of very different oscillations in one mode, or very similar oscillations in different modes. EEMD method has been developed to overcome this shortcoming of EMD. As such, in our experiments we included EEMD as well as EMD to compare their classification performance.

We have applied the proposed IMF selection approach on the classification of EEG signals recorded from epilepsy patients who are under treatment at our collaborator hospital. We have used 10-channel EEG signals recorded from 16 patients, providing a total of 160 pre-seizure, and 160 seizure (320 total) EEG segments. In addition, 4 time-domain, 5 frequency domain, and 2 nonlinear features are extracted from each selected IMF of those EEG segments. The time-domain, spectral-domain, and nonlinear features obtained from the selected three IMFs (IMF1, IMF3 and IMF2; in this order) were classified using support vector machine (SVM), K-nearest neighbor (KNN), naive Bayes, and logistic regression classifiers, and the performances of EMD and EEMD approaches were compared. Then by using this selection approach, we explore the advantages of IMF selection in either EMD or EEMD approaches as opposed to using first several IMFs (IMF1–4). In order to reveal the advantages of using EMD or EEMD approaches, the same features were extracted from the EEG signal itself, and the subbands obtained by the DWT approach, and classification processes is repeated.

Performance of SVM classifier with time feature-set was found to be poor for both approaches. When nonlinear feature-set was used, the success of four classifiers was found to be low in both approaches. Using the spectral feature-set, we obtain higher accuracies for all classifiers except logistic regression. This suggests that epileptic seizures cause distinctive changes in the frequency domain. In addition, when IMF-based classification results were evaluated, we notice that the success of classification performed only by the features obtained from the combination of selected IMFs was higher or similar to randomly selected first 4 IMFs (except nonlinear feature set). This shows that the IMF selection process helps improve the classification performance as selected IMFs carry the most useful information for the discrimination between the seizure and pre-seizure segments of EEG signals. The classification accuracy obtained using EMD or EEMD approaches using each feature-set is higher than that of the features obtained directly from EEG signals, and subbands of DWT, for all four classifiers. The computational complexity of EMD and its derivative, over classical approaches such as DWT, and fast Fourier transform (FFT) is generally considered as a disadvantage. Contrary to common knowledge, if the number of sifting steps in the EMD algorithm is equal to 10, the computational complexity is given as $\mathcal {O}(N\text{log}{N}),$ which is same as the computational complexity of FFT, where $\mathcal {O}$ denotes the order of computation, and *N* shows the signal sample size. In addition to EMD, the number of ensembles is added to the computational complexity in the EEMD approach [29]. Therefore, in signal processing applications, EMD-based approaches may be preferred considering the trade-off between the performance and computational cost.

Evaluating the channel-based classification performances, the classification success of the features obtained by EEMD approach was found to be higher than other approaches for all 4 classifiers (shown in Fig. 1).

The innovative contributions of our study can be highlighted as follows:

- We propose a hybrid IMF selection method considering different approaches such us energy, correlation, power spectral distance, and statistical significance test.
- We demonstrate the advantages of using selected IMFs by the proposed approach of either EMD or EEMD approaches as opposed to randomly selecting first several IMFs.
- We investigate the performance improvement by using ensemble EMD in the classification of epileptic seizures as compared to traditional EMD, the EEG signal itself, and DWT-based approaches.

## Conclusion

There are many studies in the literature for the detection and classification of epileptic seizures. Many studies have been performed in this field by using EMD and derivative approaches used in our study [1, 18–28]. EMD and its extensions (ensemble, multivariate and other) are suitable for the analysis of nonlinear and non-stationary signals such as EEG. In these methods, EEG signals are decomposed into IMFs which are zero–mean oscillations. Determining which of these IMFs contain useful information is vital for the success of the analysis. In most of the previous studies, the first 5 IMFs [19, 22] or first 4 IMFs [1, 17, 20, 25] have been selected, because they contain high-frequency information. In other words, no IMF selection process was performed in the initial stage of these studies. On the other hand, there are several IMF selection procedures presented in the literature based on energy, correlation coefficient, power spectrum, and statistical significance [24, 30–33]. If the signal to be analyzed contains noise, the energy and correlation coefficient of the IMFs where the noise component is dominant, will be high and misleading [30]. Therefore, the use of these IMF selection methods alone is not sufficient to determine the appropriate IMFs.

In our study, we propose a hybrid IMF selection approach considering energy, correlation, power spectral distance, and statistical significance measures. We explore the advantages of the proposed IMF selection in either EMD or EEMD approaches as opposed to using randomly selected IMFs. In our epileptic EEG classification experiments, the proposed EMD- and EEMD-based approaches outperformed the EEG-based and DWT-based approaches for all classifiers and feature sets we used. The selection algorithm for both EMD and EEMD suggests IMF1, IMF3 and IMF2 in this order. We use these IMFs separately and their combinations for feature extraction and evaluate the classification performance. The classification performance of selected IMFs and their combinations was generally higher than the classification success of randomly selected IMF1–4. It is obvious that in another signal processing problem, the selection algorithm may yield a completely different set of IMFs. Hence the use of first *k* IMFs in the classification process, as generally done in previous studies, is not the best approach. In our simulations, highest classification accuracies were obtained by using the EEMD approach where the discriminative information about epileptic seizures in the channels may be revealed more clearly (shown in Fig. 1). Note that, working with 3 or more IMFs increases both the computational load and processing time. It may be concluded that performing an IMF selection procedure before obtaining the features directly affects the success and computational load of the study.

## Materials and methods

### Proposed approach

**Fig. 2:**
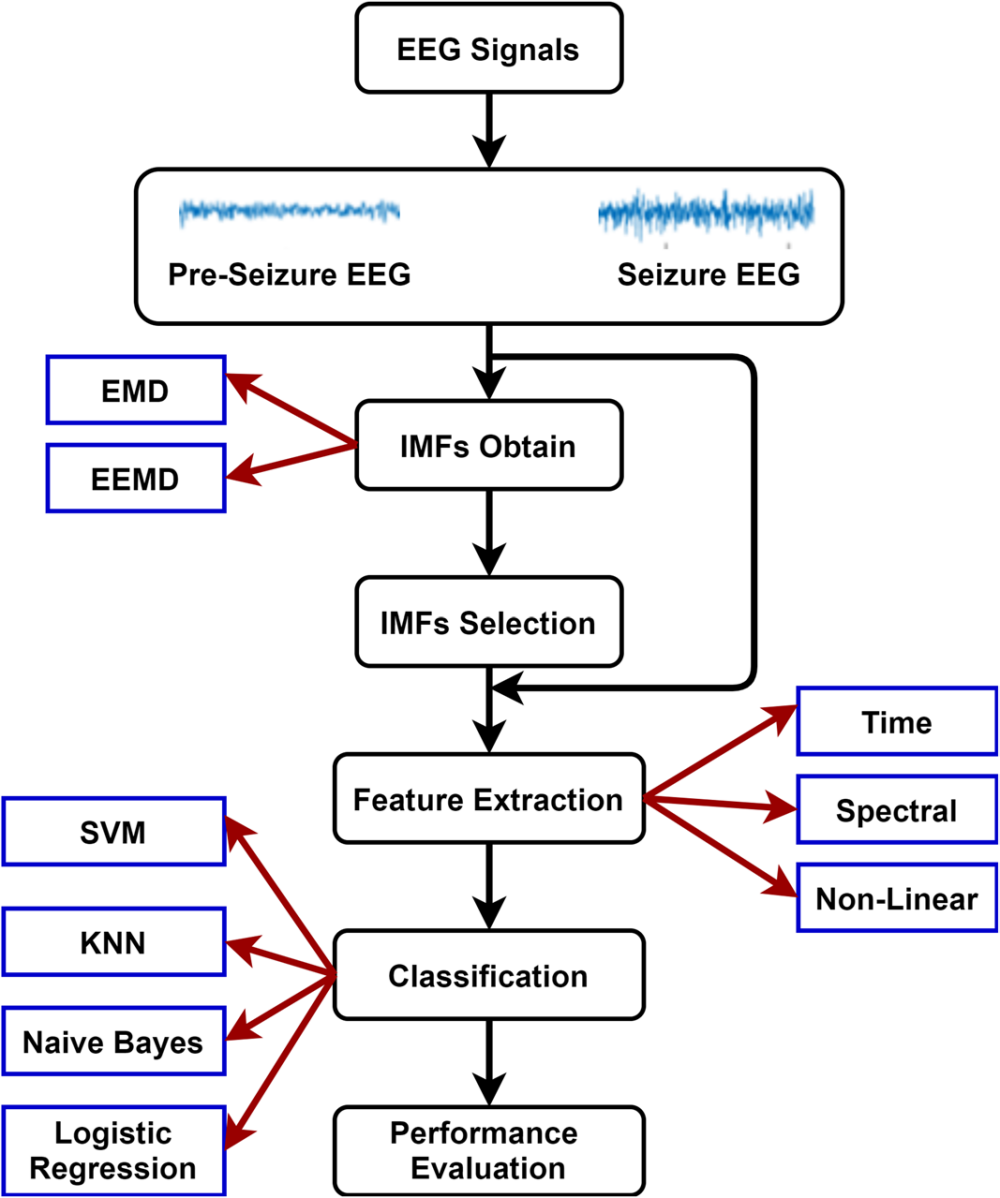
Block diagram of the proposed method

In this study, we present a method for pre-seizure and seizure classification algorithm using EMD- and EEMD-based feature extraction methods and various classifiers as depicted in Fig. 2. EEG data recorded from diagnosed epilepsy patients are labeled by physicians, and divided into pre-seizure and seizure sections. These EEG segments are decomposed into intrinsic mode functions (IMFs) using both EMD and EEMD methods for each EEG channel separately. Subsequently, optimum IMFs that best represent the signal are selected by combining several selection approaches. Following the IMF selection process, temporal, spectral, statistical and nonlinear features were calculated from the selected IMFs. Finally, the extracted features were classified by using naive Bayes, K-nearest neighbor (KNN), support vector machine (SVM), and logistic regression methods.

### Data set

Epileptic EEG data of 16 epilepsy patients recorded using surface electrodes in Izmir Katip Celebi University, School of Medicine, Neurology Department were used in this study. EEG data were recorded using the Neurofax EEG device, from 18 different channels and a sampling frequency of 100 Hz. Surface EEG data were recorded from, Fp1–F7, F7–T1, T1–T3, T3–T5, T5–O1, Fp1–F3, F3–C3, C3–P3, P3–O1, Fp2–F8, F8–T2, T2–T4, T4–T6, T6–O2, Fp2–F4, F4–C4, C4–P4, P4–O2, electrode positions, according to the International 10–20 electrode placement system. In order to use this EEG data within the scope of our study, Izmir Katip Celebi University Non-Invasive Clinical Research Ethics Committee was applied and Ethical Approval dated 08.08.2019 and numbered 296 was obtained. As discussed in [34], EEG signals recorded from the temporal and frontal lobe-weighted 10 channels (Fp1–F7, F7–T1, T1–T3, T3–T5, Fp1–F3, Fp2–F8, F8–T2, T2–T4, T4–T6, Fp2–F4) are used in the study.

One-minute pre-seizure and seizure epochs were marked by neurologist in the epileptic EEG signals recorded from selected channels. A total of 2 EEG epochs, one pre-seizure, and one seizure EEG epoch were used for each patient for our study. Thus, a total of 32 EEG epochs (containing 10 channels, for 1 min) were analyzed. Summary of the EEG data set used in the proposed study is presented in Table 5.

### Analysis of EEG signals using EMD and EEMD methods

We applied empirical mode decomposition (EMD) and ensemble EMD methods for the analysis of EEG signals in our study. In the following, we present a brief introduction to these decomposition methods.

#### Empirical mode decomposition (EMD)

Empirical mode decomposition which produces a collection of intrinsic mode functions (IMF) with zero–mean oscillations, is used as an adaptive time–frequency signal analysis method. In nonlinear and non-stationary processes, it is applied as a feature extraction and noise reduction method in signal processing applications. It is the most important rule of the EMD method that the sum of these obtained IMFs give the original signal. It is essential for the IMF to satisfy two conditions: (1) the number of zero crossing and extrema should be equal or it varies with one, (2) the mean value of the upper and lower envelopes should be zero. The process of the EMD algorithm is to extract IMF, also called Sifting, can be performed as shown in Algorithm 1 [19, 24]. 
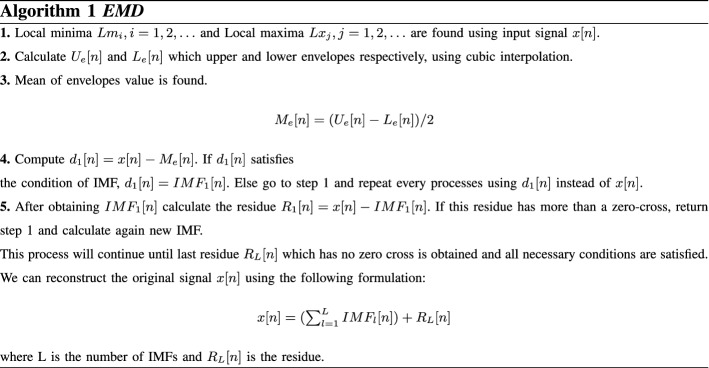


#### Ensemble empirical mode decomposition (EEMD)

Although the standard EMD algorithm provides successful results in signal processing applications as a time–frequency analysis method, it suffers from a problem called “mode mixing”. The problem of mode mixing can be described as the occurrence of very different oscillations in one mode, or very similar oscillations in different modes. The ensemble empirical mode decomposition (EEMD) method has been developed to overcome this problem. In the EEMD method, Gaussian white noise is added to the signal to be analyzed and the signal is decomposed into the intrinsic mode functions (IMF) using the EMD method. Due to the statistical properties of Gaussian white noise, the continuity of the signal is obtained in different frequency regions, so that the problem of mode mixing is reduced. The process of the EEMD algorithm is demonstrated in Algorithm 2 [28].
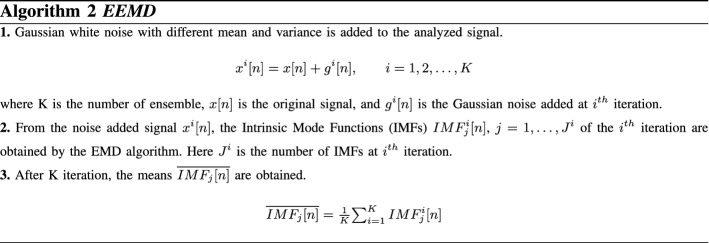


In the proposed method, we had a 10-channel and two-epoch EEG signal for each patient (total number of patients is 16). Hence the size of the pre-seizure and seizure EEG data set was 16 × 10. Maximum numbers of obtained IMFs after applying the EMD and EEMD were 16 and 15, respectively. Therefore, since it would be time-consuming and meaningless to obtain features from all IMFs, IMF selection process was performed before the feature extraction.

Discrete wavelet transforms (DWT) has widely been used for the analysis of non-stationary signals [23]. In our study, we use the DWT-based approach for feature extraction and classification of epileptic EEG segments to investigate the advantages of proposed EMD- and EEMD-based approaches. DWT decomposes a given signal *x*[*n*] into detail and approximation coefficients by using a set of mother wavelet function [23, 35]. In our study, Daubechies4 (db4) mother wavelet and 3-level subband decomposition are used.

### Selection of intrinsic mode functions (IMFs)

**Fig. 3:**
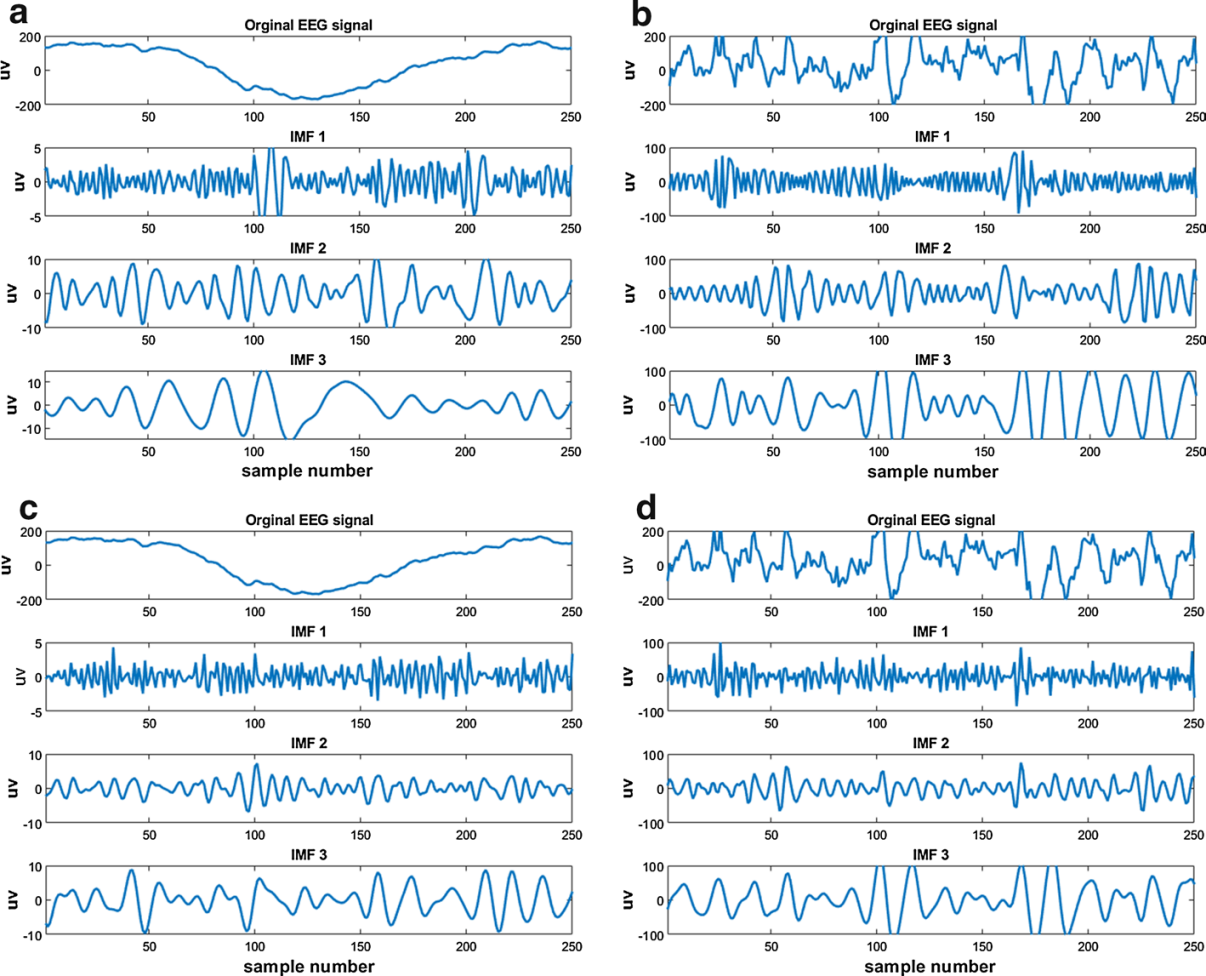
**a** Surface pre-seizure EEG signal and its first three IMF obtained using EMD; **b** surface seizure EEG signal and its first three IMF obtained using EMD; **c** surface pre-seizure EEG signal and its first three IMF obtained using EEMD; **d** surface seizure EEG signal and its first three IMF obtained using EEMD

In this study, we propose a hybrid IMF selection method by using energy-based, correlation-based, PSD distance-based, and t-test-based approaches. Pre-seizures and seizures epileptic EEG data of 16 patients recorded from 10 channels were decomposed into the IMFs using both EMD and EEMD approaches (example signals are shown in Fig. 3), then the proposed IMF selection procedure in the following described is executed.

#### Energy-based selection method

The energies of each IMFs are calculated as shown in Eq. (1). Since the higher-energy IMF is considered to be the best representative of the original signal, the IMFs were ranked from the high to the low energy IMF [30].

1 $$E_{\text{IMF}_{i}}=\sum _{n=0}^{N-1}|\text{IMF}_{i}[n]|^{2}, \quad i= 1,\ldots ,L.$$

Here, $\text{IMF}_{i}$ is the $i{\text{th}}\;{\text{IMF}}$ and $E_{\text{IMF}_{i}}$ is the energy of this IMF.

#### The correlation-based selection method

The correlation coefficient of each IMFs are calculated as shown in Eq. (2). Since the IMF with high correlation coefficient is considered to be a good representative IMF of the original signal, the IMFs are ranked from the high to low correlation coefficient IMF [31].

2 $$\rho _{x, \text{IMF}_{i}}=\frac{C_{x,\text{IMF}_{i}}}{\sigma _{x}\sigma _{\text{IMF}_{i}}}.$$

Here, $C_{x,\text{IMF}_{i}}$ is the cross-covariance of the original signal and $i{\text{th}}\;{\text{IMF}}$, $\sigma _{x}$, and $\sigma _{\text{IMF}_{i}}$ are the standard deviations of the original signal and $\text{IMF}_i,$ respectively, and$\rho$ denotes the correlation coefficient.

#### The PSD distance-based selection method

Another IMF selection method, based on power spectral densities (PSD) was also utilized by using the power spectral densities of the original signal and IMFs. The distances between the estimated PSDs are calculated using the Kullback Liebler distance (KLD) method as shown in Eq. (3). If the distance between the PSDs of original signal and an IMF is minimum, that IMF is considered to be the best representative IMF of the original signal. Hence, the IMFs are ranked from the low to the high PSD distance IMF [32, 33].

3 $${\text{dis}}_{\text{KLD}}(x,{\text{IMF}}_{i})=\sum _{n=0}^{N-1}\log \frac{S_x(\omega _k)}{S_{\text{IMF}_{i}}(\omega _k)}, \quad \omega _k= \frac{2\uppi }{N}k,$$

where $S_x(.)$ is the power spectrum of the original signal, $S_{\text{IMF}_{i}}(.)$ is the power spectrum of the $i{\text{th}}\;\text{IMF}$, the ${\text{dis}}_{\text{KLD}}(x,\text{IMF}_{i})$ shows the KLD between the power spectra of the $i{\text{th}}\;{\text{IMF}}$ and that of the original signal.

#### Statistical significance-based selection method

We also use the *t*-test statistical significance measure for the selection of best IMFs. The *t*-test is based on the principle of generating a null hypothesis that a single sample data set comes from a normal distribution. In this statistical significance test, test statistic values; *h*-value and *p*-value are calculated. Here, the *h*-value indicates whether the distribution of data is normal, and the *p*-value indicates the statistical significance of the data. If a *p*-value greater than the specified threshold of $\alpha$ (often chosen as 0.05 or 5% in the literature), the distribution of data can be interpreted as normal (null hypothesis is satisfied, *h*-value = 0). Otherwise, if this *p*-value is less than that threshold, the distribution of data may not be interpreted as normal (null hypothesis is not satisfied, *h*-value = 1). The *p*-values of the data whose distribution is known to be normal (*h*-value = 0) can be used as a statistical significance measure. It has previously been recommended to select the IMFs with high *p*-values in order to create a feature set with improved classification performance [24]. As such, we calculate the *p*-value for every IMFs by applying the *t*-test. Since the *p*-value obtained here shows the statistical significance of IMFs, the IMFs are ranked from the high to low *p*-value IMF.

Table 6 shows the results of the above four selection approaches for one of the patients and one EEG channel.

**Table 6:** Example of IMF ranking matrix

Component	Order of IMF											
	$1{\text{st}}$	$2{\text{nd}}$	$3{\text{rd}}$	$4{\text{th}}$	$5{\text{th}}$	$6{\text{th}}$	$7{\text{th}}$	$8{\text{th}}$	$9{\text{th}}$	$10{\text{th}}$	$11{\text{th}}$	$12{\text{th}}$
Energy	7	6	8	5	9	10	4	1	3	2	11	12
Correlation coefficient	7	6	8	9	5	4	10	3	11	12	1	2
PSD distance	1	2	3	4	5	6	7	8	9	10	11	12
*p* value	3	2	1	7	4	9	5	6	10	8	11	12

Here, $7{\text{th}}$ IMF has the highest energy while $12{\text{th}}$ IMF has the lowest energy.

$7{\text{th}}$ IMF has the highest correlation coefficient while $2{\text{nd}}$ IMF has the lowest correlation coefficient.

$1{\text{st}}$ IMF has the lowest PSD distance while $12{\text{th}}$ IMF has the highest PSD distance.

$3{\text{rd}}$ IMF has the highest p value while $12{\text{th}}$ IMF has the lowest p value.

Each row shows the ranking of the obtained IMFs according to that features

**Fig. 4:**
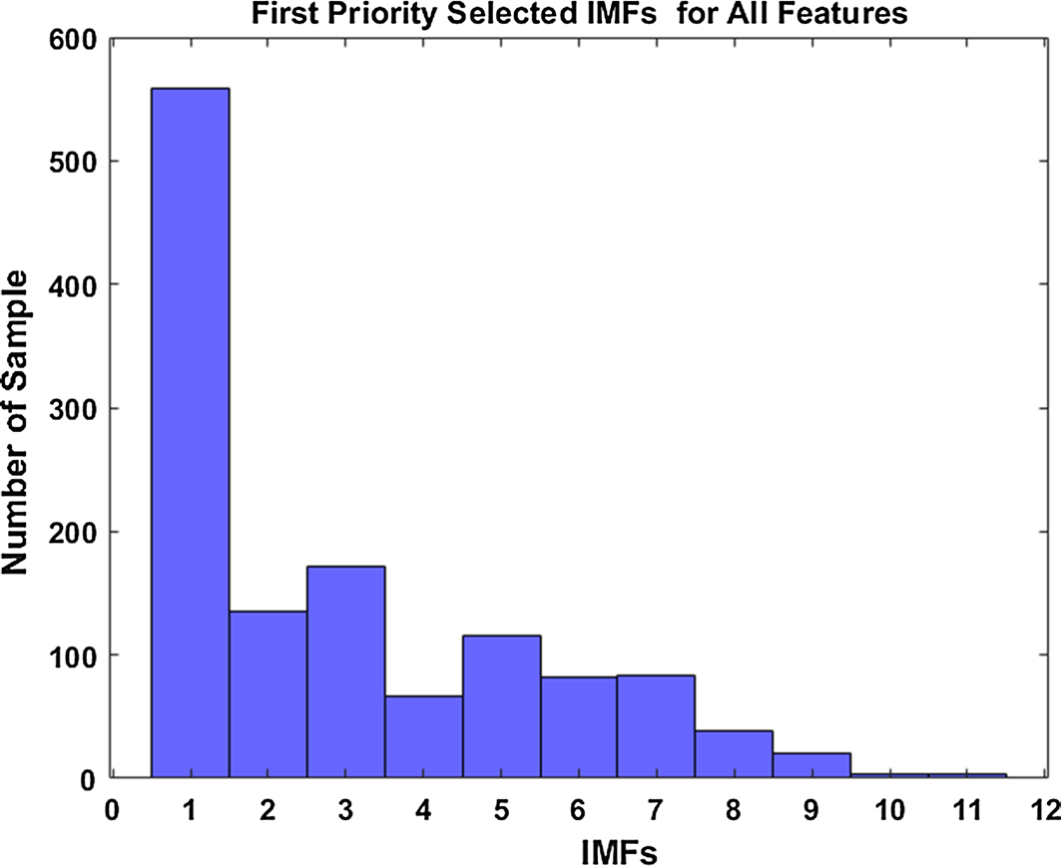
Histogram of first priority selected IMFs

These procedures were applied to the pre-seizure and seizure EEG data of 10 different channels of each patient separately. Finally, 40 metrics for 10 channels are calculated for each patient. All ranking matrices were combined and a 1280 × 16 -dimensional ranking matrix for all pre-seizure and seizure EEG data was obtained. To determine the first priority selected IMFs for all signals, the histogram of the 1st column of the ranking matrix was calculated. The resulting histogram is shown in Fig. 4.

Examining the histogram shown in Fig. 4, we observe that the IMF1 is the first priority selected IMF, IMF2 is the third, and IMF3 is the second priority selected IMF. In our simulation, we choose these three IMFs (IMF1, IMF3, IMF2) for feature extraction.

The histogram shown in Fig. 4 suggests IMF1, IMF3 and IMF2 in this order.

### Classification of pre-seizure and seizure EEG segments

In this section, we present a method to classify the pre-seizure and seizure segment of EEG signals collected from epilepsy patients. These EEG signals are detailed introduced in "Data set" section. We use the selected best IMFs represented the EEG signals, we extract a set of feature.

#### Feature extraction

Time-domain, spectral, and nonlinear features were obtained using the selected IMFs and original EEG signals to obtain feature sets.

- Time-domain feature set: after the IMF selection process was carried out, the time-domain feature data set was created, using directly the EEG signals, using the first three of the IMFs obtained by EMD and EEMD methods, and using the subbands of DWT. Energy, mean value, skewness, and kurtosis values were calculated for 3 IMFs, DWT subbands, and EEG signals in the time-domain [8, 23].
  4 $$E= \sum _{n=0}^{N-1}|X[n]|^{2}$$
  5 $$\mu=\frac{1}{N}{\sum _{n=0}^{N-1}{X[n]}}$$
  6 $$S = \frac{\frac{1}{N}{\sum _{n=0}^{N-1}\left(X[n]-\mu \right)^{3}}}{\left(\sqrt{\frac{1}{N}{\sum _{n=0}^{N-1}\left([n]-\mu \right)^{2}}}\right)^3}$$
  7 $$K= \frac{\frac{1}{N}{\sum _{n=0}^{N-1}(X[n]-\mu )^{4}}}{\left(\frac{1}{N} {\sum _{n=0}^{N-1}(X[n]-\mu )^{2}}\right)^2}$$

In the above equations, *X*[*n*] indicates the EEG signal or IMFs, *N* is the size of the signal or IMFs. *E* denotes the energy, $\mu$ is the mean value; *S* indicates the skewness, *K* is the kurtosis value.

In the EMD- and EEMD-based approaches a total of 320 × 12 size, and DWT -based approach a total of 320 × 16 size feature sets were obtained. Applying the same procedure to the EEG signal itself, a total of 320 × 14 size feature set for pre-seizure and seizure EEG data was obtained.

- Spectral-domain feature set: to generate this feature data set, the spectrum of the signal or IMF calculated by the periodogram method was used. Total power, spectral entropy, 1st, 2nd, and 3rd moments were calculated using the spectrum of signals [10, 26].
  8 $$S(\omega _k) = \frac{1}{N}|X(\omega _k)|^{2}$$
  9 $$S_{\text{T}}= \sum _{k=0}^{N-1}{S(\omega _k)}$$
  10 $$M_j= \sum _{k=0}^{N-1}(\omega _k)^jS(\omega _k), \quad j= 1,2,3$$
  11 $$H= -\sum _{k=0}^{N-1}{P(\omega _k)\log _2{P(\omega _k)}}$$

Here, in Eqs. (8) and (9), $S(\omega _k)$ denotes the power spectral density of the signal estimated by periodogram method, $X(\omega _k)$ is the discrete Fourier transform of the signal *x*[*n*] [10], and $S_{\text{T}}$ is the total power. In addition, *N* indicates the size of the corresponding signal and $\omega _k= \frac{2\uppi }{N}k$; $M_j$ given in Eq. (10), indicate the higher order spectral moments of the corresponding signal. *H* shown in Eq. (11) denotes the spectral entropy of the signal, and $P(\omega _k)=\frac{S(\omega _k)}{S_{\text{T}}}$ indicates the normalized power spectral distribution [26].

In the EMD- and EEMD-based approaches a total of 320 × 15 size, and DWT -based approach a total of 320 × 20 size feature sets were obtained. Applying the same procedure to the EEG signal itself, a total of 320 × 5 size feature set for pre-seizure and seizure EEG data was obtained.

- Nonlinear feature set: nonlinear features such as the Hurst exponent and Higuchi fractal dimension were computed to obtain this feature data set. These nonlinear features are used to analyze the complexity and self-similarity of brain recordings and other biological signals. Calculation of Hurst exponent and Higuchi fractal dimension were given in Eqs. (12), (13), (14), and (15); (16), (17), and (18), respectively.
  12 $$X[n]= \{X[1], X[2],\ldots , X[N]\}$$
  13 $$X_\text{A}[n]= \sum _{i=1}^n{X[i]-\mu }, \quad n=1,\ldots ,N$$
  14 $$\begin{aligned} R[m] & = \text{max}(\{X_A[1], X_{\text{A}}[2],\ldots , X_{\text{A}}[m]\})-\text{min}(\{X_{\text{A}}[1], X_{\text{A}}[2],\ldots ,X_{\text{A}}[m]\}), \\ S[m] & = \sqrt{\frac{1}{m}{\sum _{k=1}^m(X[k]-\bar{X}_m})^{2}},\quad m = 1,\ldots , N \end{aligned}$$
  15 $${\text{LN}}= \ln {\frac{R(k)}{S(k)}},\quad k = 1,\ldots , N,$$
  where *X*[*n*] given in Eq. (12) shows the EEG signal or the IMFs to be analyzed and $\mu$ indicates the mean value of this signal. The $X_{\text{A}}[n]$ shown in Eq. (13) indicates the accumulated deviation value of *X*[*n*]. Equations (14) shows the range series *R*[*m*] and the standard deviation *S*[*m*] of the time-series *X*[*n*], and $\bar{X}_m$ is the mean value from *X*[1] to *X*[*m*]. In Eq. (15), LN shows the logarithmic value. The Hurst exponent is calculated as the slope of the line where LN is plotted with respect to ln(k).

The value of Hurst Exponent (HE) ranges from 0 to 1. If there is no correlation in the time-series, $\text{HE} =0.5$; if time-series has long-range anti-correlations, $0< \text{HE} < 0.5$ and if there is long-range correlations in the time-series, $0.5< \text{HE} < 1$ [16].

Higuchi fractal dimension (HFD) used to calculate the fractal dimension (FD) directly from time-series signals. The most important parameter that must be determined for the calculation of Higuchi fractal dimension is $k{(\text{max})}$. The HFD values calculated in a given $k{(\text{max})}$ range are plotted against this range in order to determine the optimal value for the $k{(\text{max})}$ parameter. The k value that the obtained curve reaches the saturation point is determined as $k{(\text{max})}$ [17, 36].

16 $$\begin{aligned} X & = \left\{ {X[1],X[2], \ldots ,X[N]} \right\} \\ {X_{k}}^m & = \left\{ {X[m],X[m + k],X[m + 2k], \ldots ,X\left[ {m + {\text{int}}\left( {\frac{{N - M}}{k}} \right)*k} \right]} \right\}, \\ m & = 1,2, \ldots ,k. \end{aligned}$$

17 $$L[m,k] = \frac{{\left\{ {\left( {\sum\limits_{{i = 1}}^{{\text{int}\left( {\frac{{N - M}}{k}} \right)}} {\left| {X[m + ik] - X[m + (i - 1)k]} \right|} } \right)\frac{{N - 1}}{{\text{int}\left( {\frac{{N - M}}{k}} \right)}}} \right\}}}{k}$$

18 $$L[k]= \frac{1}{k}{\sum _{m=1}^k{L[m,k]}}, \quad m=1, 2,\ldots ,k.$$

In Eq. (16) , *X* indicates the one-dimensional time-series EEG signal or the IMFs and ${X_{k}}^m$ indicates the new time-series. Here, *k* and *m* are integers and the int(.) operation indicates the integer part of the $(N-m)/k$ value, *N* is the length of the original signal. The *L*[*m*, *k*] calculated in Eq. (17) indicates the the size of the new time-series signals. The *L*[*k*] calculated by using the average of the *L*(*m*, *k*) values in Eq. (18) indicates the length of the curve for the *k* new time interval. HFD is calculated as the slope of the line where *L*[*k*] is plotted with respect to $\text{ln}[1/k],k=1,2,\ldots ,k_{\text{max}}$.

In our study, HFD values calculated against different *k* (max) values were plotted and a graph was obtained. It was observed that this graph reached saturation point when $k_{\text{max}} = 30.$

In the EMD- and EEMD-based approaches a total of 320 × 6 size, and DWT -based approach a total of 320 × 8 size feature sets were obtained. Applying the same procedure to the EEG signal itself, a total of 320 × 2 size feature set for pre-seizure and seizure EEG data was obtained.

#### Classification

Features extracted from the selected IMFs of the EEG signals are used to discriminate the pre-seizure and seizure segments of the EEG by using the support vector machines (SVM), K-nearest neighbor (KNN), and naive Bayes classifiers. In the following, we present the fundamentals of these classification methods.

- Support vector machine (SVM): support vector machine (SVM), a supervised machine learning algorithm, is a successful algorithm that is frequently used in both classification and regression studies. In this algorithm, the elements of the data set containing *n* features are placed as elements of the coordinate system in an *n*-dimensional space. Then, the classification is performed by finding the hyperplane that separates the classes best. There are many possible hyperplanes that can separate the two classes. What is important here is to choose the hyperplane from which the highest classification performance may be achieved.

Let $(x_k,y_k)$ be given as a separable sample example. Here, *k* indicates the size of the feature set and $y\in \{-1,1\}$ indicates the class label. Thence, separating hyperplane can be formulated with $f(x)=\vec{w}x+c.$ Here, $\vec{w}$ indicates the hyperplane parameters and *c* indicates the offset. The hyperplanes that can separate the two classes from each other with minimum error provide $y_k[(\vec{w}x_k)+c]-1\ge 0, k=1, 2,\ldots , n$ condition. The main purpose here is to achieve the maximum margin. Here, the margin is the distance between the support vectors belonging to two different class. Finally, the data falling on different sides of the hyperplane is assigned as an element of a different class [13, 14, 18, 19, 26].

- K-nearest neighbor (KNN): it is one of the learning-based pattern recognition methods. The data set is divided into two parts as training and tests then the learning process is performed according to the data in the training set. First, the distance between the sample to be classified and all the data in the training set is calculated. Then, the K-nearest neighbors that have minimum distance is determined. Finally, the most common class among these K-nearest neighbors is selected as the class of the new sample. Various distance measurement methods such as Euclidean, Manhattan, Minkowski, and Hamming can be used for distance calculation [26, 35, 37]. In our study, the most commonly used Euclidean distance calculation method is used [shown in Eq. (19)] and *k* value is chosen as 5.
  19 $$\text{ED}=\sqrt{\sum _{m=1}^n{(x_m-y_m)^2}}$$
- Naive Bayes: it is one of the probabilistic classifier based on Bayes theorem in which classification is performed according to probability basics. The classification process is performed by calculating the membership probability of a sample to all classes in the data set.

Let $X=\{x_1,x_2,\ldots ,x_n\}$ be given. Here, n is the number of features, *X* indicates the sample in the feature-set. In addition, $\{M_1, M_2,\ldots ,M_m\}$ represents classes, here m is the number of classes. The probability that each *X* data in the data set is a member of the $M_i$ class is calculated as given in Eq. (20):

20 $$\begin{aligned}& P(M_i/X)=\frac{P(X/M_i)P(M_i)}{P(X)} \\& \text{if}; \qquad P(M_i/X)>P(M_j/X),\quad 1\le j\le m, \quad j\ne i. \end{aligned}$$

Then the *X* data is assigned to the class in which class membership is highest. Here, *X* data is assigned to the $M_i$ class, where $P(M_i)$ indicates the class prior probabilities, *P*(*X*) indicates the prior probability of sample *X*, $P(X/M_i)$ indicates the probability of *X* conditioned on $M_i$ and $P(M_i/X)$ indicates the probability of $M_i$ conditioned on *X* [35, 37].

- Logistic regression: logistic regression (LR) is a frequently used statistical classification technique in which the probability (*P*1), of dichotomous outcome event limited to two values such as yes/no, on/off, or 1/0, is related to a set of independent variables, and given in Eq. (21):
  21 $$\text{logit}(P_1)= \ln \left( \frac{P_1}{1-P_1}\right) =\beta _0+ \beta _1X_1+ \ldots + \beta _nX_n.$$
  Here, $\beta _0$ is the intercept and $\{\beta _1X_1+ \cdots + \beta _nX_n\}$ are the coefficients associated with the independent variable $\{X_1, X_2,\ldots , X_n\}$. Generally, in the logistic regression method, the maximum likelihood estimation (MLE) method is used to calculate the coefficients $\{\beta _1X_1+ \cdots + \beta _nX_n\}$.

The probability of an event existing as a function of the independent variables is nonlinear as extracted from Eq. (22) [38]:

22 $$P_1(X)= \frac{P_1}{1+e^{-{\text{logit}}(P_1(X))}}.$$

Here, $P_1\in \{0,1\}$ indicates the probability value.

If the result of our Eq. (22) is $-\infty$, the probability is 0 ($P_1=0$), and if the result of this equation is $\infty$, our probability is 1.

#### Performance evaluation

In this study, accuracy (ACC), sensitivity (SEN), selectivity (SPE), and precision (PRE) expressed as the performance criteria and *F*-score values that is the combination of previous parameters were used for performance evaluation. Fivefold cross-validation (CV) method has been used to establish the performances of the classifiers.

The feature set used in the k-fold CV method is randomly separated into k different folds with the same size. Of these *k* folds, (*k* − 1) folds are used for training and the other one (1) fold is used for testing. No fold is used for validation processes. This process is repeated *k* times and the accuracy value is calculated separately for each iteration. After *k* iterations, the average accuracy value is obtained. This average accuracy obtained is accepted as CV accuracy [21, 23].

23 $$\begin{aligned} \text{ACC} & = \frac{\text{TP}+\text{TN}}{\text{TP}+\text{FN}+\text{FP}+\text{TN}}*100{\%} \\ \text{SEN} & = \frac{\text{TP}}{\text{TP}+\text{FN}}*100{\%} \\ \text{SPE} & = \frac{\text{TN}}{\text{FP}+\text{TN}}*100{\%} \\ \text{PRE} & = \frac{\text{TP}}{\text{TP}+\text{FP}}*100{\%} \\ F_{\text{score}}& =2*{\frac{\text{PRE}*\text{SEN}}{\text{PRE}+\text{SEN}}}*100{\%} \end{aligned}$$

In Eq. 23 , false-positive (FP) indicates the number of samples for class 0, but is mistaken for class 1 by the algorithm. False-negative (FN) denotes the number of samples for class 1, but is mistaken for class 0 by the algorithm. True-positive (TP) (the number of samples for class 1) and true-negative (TN) (the number of samples for class 0) indicate the numbers of samples that are exactly classified by the algorithm [13, 14].

## Data Availability

The datasets generated during and/or analyzed during the current study are available from the corresponding author at reasonable request.

## Abbreviations

EEG: Electroencephalogram
EMD: Empirical mode decomposition
EEMD: Ensemble empirical mode decomposition
IMF: Intrinsic mode function
SVM: Support vector machine
KNN: K-nearest neighbors
